# C-reactive Protein Is a Diagnostic Tool for Postoperative Infection in Orthopaedics

**DOI:** 10.7759/cureus.22270

**Published:** 2022-02-16

**Authors:** Saiganesh Shetty, Prabhu Ethiraj, Arun H Shanthappa

**Affiliations:** 1 Orthopaedics, Sri Devaraj Urs Academy Of Higher Education and Research (SDUAHER), Kolar, IND

**Keywords:** diagnostic tool, fracture surgeries, postoperative infection, trauma, crp

## Abstract

Background

Orthopedic fracture-associated infection is a prevalent complication with a huge burden on the healthcare infrastructure. C-reactive protein (CRP) is a widely used serum inflammatory marker in patients with infections in orthopaedics. It could be difficult to distinguish between CRP elevation caused by surgical site infection and CRP elevation caused by surgery and trauma in orthopaedic procedures. In most situations, a clinical diagnosis of post-surgical infection is sufficient, however, the use of a biomarker with predictive value for acute post-op complications could prompt an earlier diagnosis. This study, therefore, aims at assessing CRP levels in post-operative orthopaedic trauma patients and determining the reliability of CRP as an early indicator of postoperative infection.

Materials and methods

A prospective study was conducted between December 2020 and November 2021 in the department of orthopaedics in Sri Devaraj Urs medical college, Kolar. Patients with an open and closed fracture of the upper and lower extremities treated by osteosynthesis on an elective or emergency basis were included. The clinical parameters were studied on the day of trauma, postoperative days first, third and seventh. Blood samples for CRP were taken prior to the surgical procedure and on the same days as clinical monitoring. The CRP levels were compared between patients with postoperative infection and patients without postoperative infections using independent samples t-test. A p-value of < 0.05 was considered statistically significant.

Results

A total of 51 patients were included in the study meeting the inclusion criteria, of which mean standard deviation for age was 37.5 (15.7%), 44 were men (86.2%) and seven were women (13.7%), Patients according to Tscherene classification grade I were 10 (19.6%), grade II were eight (15.6%), grade III were 15 (29.4%) and grade IV was 18 (35.2%), type of fracture surgery diaphyseal were 27 (52.9%), proximal was 11 (21.5%) and distal were 13 (25.4%). 15 patients developed postoperative infection with CRP levels of 96 µg/mL in nine (17.6%), 48 µg/mL in four (7.8%) and 24 µg/mL in two (3.9%). Thirty-six patients who did not develop post-operative infection had CRP levels of 6 µg/mL in 31 (60.8%) and 12 µg/mL in five (9.8%). The p-value for the first postoperative day was 0.289 and statistically insignificant and on the third and seventh postoperative days was <0.001 and was found to be statistically significant.

Conclusion

C-reactive protein is a useful parameter to detect and monitor post-operative infections in orthopaedic trauma surgeries. The rise in C-reactive protein on the third and seventh postoperative days can be used as a reliable predictor of post-operative infections.

## Introduction

Post-operative wound infection is estimated to have an incidence of about 3.3 % following internal fixation in fracture management (osteosynthesis) [1]. Surgical site infections (SSIs) do have a wide range of risks, with orthopaedic trauma having a significant rate of SSIs than many other surgical specialties [2]. Fracture-related infections (FRI) contribute significantly to morbidity amongst patients accounting for almost 30% of postoperative complications in this patient population [3]. Sequelae of infection can lead to permanent functional loss or amputation of the affected limbs and reduced quality of life [4]. Early diagnosis of infections and initiation of appropriate treatment protocol following the surgical intervention is critical in the prognosis and preventing its complications. The early diagnosis of post-surgical infection is mostly clinical. However, laboratory investigations aid in the diagnosis of acute infectious complications following surgical intervention for fracture. C-reactive protein (CRP) is a widely recognized serum inflammatory marker in patients with infections.CRP is secreted by the liver in response to multiple stimuli [5]. CRP is an acute-phase protein with a normal level below 6 µg/ml. It has a short biological half-life with negligible circadian variation [6,7]. Elevated CRP levels following surgery are a normal phenomenon. The doubling time of CRP in response to a stimulus is approximately eight hours [8]. Deviations from the normal rise in CRP may indicate an infection and diagnose surgical complications at an early stage. It could be difficult to distinguish between CRP elevation caused by surgical site infection and CRP elevation caused by surgery and trauma in orthopaedic procedures. In most situations, a clinical diagnosis of post-surgical infection is sufficient, however diagnostic testing is required to predict acute infectious complications following fracture surgery. This study aims at assessing CRP level in post-operative orthopaedic trauma patients and determining the reliability of CRP as an early indicator of postoperative infection.

## Materials and methods

A prospective study was conducted between December 2020 and November 2021 in the department of orthopaedics in Sri Devaraj Urs Medical College, Kolar. Prior to the start of the study, Sri Devaraj Urs Medical College's institutional ethical committee clearance was obtained. The research approval number is SDUMC/KLR/IEC/49/2019-20. Patients with an open and closed fracture of the upper and lower extremities treated by osteosynthesis on an elective or emergency basis were included. Tscherene classification was used since it incorporates both closed and open fractures and is classified from grades I-IV grades. Patients with other concomitant injuries at the time of presentation like solid organ injuries, genitourinary injuries, and pulmonary injuries were excluded from the study. Body temperature/fever, local hyperthermia, skin necrosis, abscess, or purulent discharge from the surgical site were defined as clinical criteria for the diagnosis of infection complications prior to the start of the study. When a discharge or abscess occurred, bacteriological samples are collected. On the day of the trauma, post-operative days first, third and seventh, the aforementioned parameters were investigated. On the day of the trauma and on the same days as clinical surveillance, blood samples for CRP were collected through venepuncture. CRP levels above > 10 µg/mL were considered abnormal. Even if bacteriological samples were negative, a clinical diagnosis of infection was made based on the macroscopic presence of pus, either from the traumatic or surgical lesion.

The sample size was estimated using the mean and standard deviation of CRP during the post-operative period was reported to be 77.3 ± 53.3 mg/L [9] by Bourguignat et al. The expected population standard deviation is 53.3, and employing t-distribution to estimate sample size, the study would require a sample size of 51 subjects to estimate the mean CRP levels with 95% confidence and a precision of 15 mg/L. Data obtained was entered using Microsoft Excel and analyzed using the Statistical Package for Social Science (SPSS) (IBM Corp. Released 2011. IBM SPSS Statistics for Windows, Version 20.0. Armonk, NY: IBM Corp.). All continuous variables were summarised using mean (SD) or median (IQR) depending on the normality of the distribution. Categorical variables were summarised using proportions. Normality was assessed using the Shapiro-Wilks test and homogeneity of variance was checked using Levene’s test. The CRP levels were reported using Mean along with SD. The CRP levels between those who have post-operative infections and those who do not have post-operative infections were compared using an independent samples t-test. Comparison of categorical variables across study groups was done using the Chi-square test. A p-value of < 0.05 was considered statistically significant.

## Results

A total of 51 patients were included in the study meeting the inclusion criteria, of which mean standard deviation for age was 37.5 (15.7%), 44 were men (86.2%) and 7 were women (13.7%), Tscherene classification grade I were 10 (19.6%), grade II were eight (15.6%), grade III were 15 (29.4%) and grade IV were 18 (35.2%), 27 (52.9%) patients underwent elective surgery and 24 (47%) patients underwent emergency surgery, Location of fracture surgery diaphyseal were 27 (52.9%), proximal were 11 (21.5%) and distal were 13 (25.4%) (Table 1).

**Table 1:** Clinical and demographic characteristics SD - Standard deviation

Characteristics	Frequency (%)
Mean (SD) age	37.5 (15.7)
Age groups	
17 – 30 years	23 (45.1%)
31 – 45 years	12 (23.5%)
46 – 60 years	10 (19.6%)
61 – 75 years	6 (11.8%)
Gender	
Male	44 (86.3%)
Female	7 (13.7%)
Tscherne classification	
Grade I	10 (19.6%)
Grade II	8 (15.6%)
Grade III	15 (29.4%)
Grade IV	18 (35.2%)
Type of surgery	
Elective	27 (52.9%)
Emergency	24 (47%)
Location of the fracture site	
Diaphyseal	27 (52.9%)
Distal	11 (21.5%)
Proximal	13 (25.4%)

From a total of 51 patients, 15 patients had developed postoperative infection with CRP levels of >96 µg/mL in nine (17.6%), 46-95µg/mL in four (7.8%) and 21-45 µg/mL in two (3.9%). 36 patients who did not develop post-operative infection had CRP levels of < 10µg/mL in 31 (60.8%) and 10-20 µg/mL in five (9.8%) (Table 2). 15 patients with discharge from the surgical site were sent for isolation of organism, in all patient’s organism isolated was *Staphylococcus Aureus*.

**Table 2:** Comparison of C reactive protein levels and clinical signs of infections on different clinical monitoring days POD - Postoperative day, CRP - C reactive protein

	Preoperative	POD 1	POD 3	POD 7
CRP levels				
<10 µg/mL	4 (7.8%)	9 (17.6%)	25 (49.0%)	31 (60.8%)
10-20 µg/mL	43 (84.3%)	28 (54.9%)	11 (21.6%)	5 (9.8%)
21-45 µg/mL	4 (7.8%)	11 (21.6%)	4 (7.9%)	2 (3.9%)
46-95 µg/mL	-	3 (5.9%)	11 (21.6%)	4 (7.8%)
>96 µg/mL	-	-	-	9 (17.6%)
Body temperature (>99 F)	5 (9.8%)	5 (9.8%)	11 (21.6%)	14 (27.4%)
Local Hyperthermia	0	2 (3.9%)	12 (23.5%)	15 (29.4%)
Skin necrosis	0	2 (3.9%)	10 (19.6%)	15 (29.4%)
Abscess/discharge from surgical site	0	2 (3.9%)	9 (17.6%)	15 (29.4%)
Presence of infection	-	7 (13.7%)	14 (27.4%)	15 (29.4%)

From a total of 15 patients who had an infection, nine patients were operated on an emergency basis and six were operated on an elective basis. A multivariate test (Wilks' lambda) was used and the p value of 0.181 or below was found to be statistically significant. P-value for postoperative day one was 0.289 and statistically insignificant and postoperative days three and seven were < 0.001 and was found to be statistically significant (Table 3). 

**Table 3:** Comparison of C reactive protein levels, presence of infection and Multivariate logistic tests CRP - C reactive protein, PRE OP - Pre operative, POD - Post operative day

Monitoring days	Infection	Mean CRP values (mg/ml)	Standard deviation	Number of patients	Multivariate test	p-value
PRE OP	Absent	12.72	2.804	36	Wilks' Lambda	0.356
PRE OP	Present	16.93	6.798	15	Wilks' Lambda	0.356
PRE OP	Total	13.96	4.712	51	Wilks' Lambda	0.356
POD 1	Absent	12.00	3.928	36	Wilks' Lambda	0.289
POD 1	Present	29.73	13.588	15	Wilks' Lambda	0.289
POD 1	Total	17.22	11.362	51	Wilks' Lambda	0.289
POD 3	Absent	8.31	3.592	36	Wilks' Lambda	<0.001
POD 3	Present	51.60	17.808	15	Wilks' Lambda	<0.001
POD 3	Total	21.04	22.243	51	Wilks' Lambda	<0.001
POD 7	Absent	7.39	2.697	36	Wilks' Lambda	<0.001
POD 7	Present	80.33	25.195	15	Wilks' Lambda	<0.001
POD 7	Total	28.84	36.189	51	Wilks' Lambda	<0.001

## Discussion

CRP is a commonly used inflammatory marker in the blood that is used to assess infection. In our current investigation, we established that an increase in CRP levels and the presence of postoperative wound infection in patients receiving internal fixation (osteosynthesis) in fracture care was statistically significant. Patients with elevated CRP levels were found to have a post-operative wound infection on the third and seventh postoperative days. The preoperative CRP value is required to identify some pre-existing illnesses; nevertheless, the preoperative CRP value is known to rise after a fracture. Prior to the surgery, CRP levels were found to be higher in all participants in our study. The primary trauma can be implicated for the elevation in CRP levels considering trauma is a known inciting incident that triggers an inflammatory response. The peak rise of CRP levels was seen on the third post-operative day, this observation is consistent with results from other studies, with maximal responses achieved either on the second postoperative day or the third post-operative day. CRP levels above 96 mg/mL on or after the fifth postoperative day were found to have a sensitivity of 92 percent and a specificity of 93 percent in detecting deep wound infections following limb fracture surgery in previous studies. A CRP result of ≥ 140 mg/ml from the fourth post-operative day indicated post-operative infection, according to another study [10,11]. The normal pattern of CRP rise is only until the third postoperative day, i.e., it rises until the third postoperative day and then reverts into the normal range [12]. A multivariate test (Wilks lambda test) was used for statistical analysis to know the significance of CRP at different timelines. It showed that CRP values present on postoperative days third and seventh were statistically significant. In our study, individuals who did not develop postoperative wound infection exhibited a similar trend. It was observed that postoperative wound infections had higher mean CRP values on the third and seventh postoperative days than those who did not have a postoperative infection.

Surgical intervention triggers an immunological response, which is mediated by cytokines [13]. In a study conducted by Larrson et al., they concluded that age, sex, operation time, amount of bleeding, blood transfusion, pre/post-operative drugs, and kind of anesthesia have little effect on CRP levels [14]. In a study conducted by Neumaier et al., they observed that increase in CRP is rather linked to the surgical technique, fracture site, and surgical trauma. CRP levels rose the most in femur fractures, according to various investigators [15,16]. A high CRP level is a well-known predictor of poor outcomes in elderly patients undergoing vascular and oncosurgery [17,18].

Chitnis et al. reported that patients with infection had significantly higher rates of hospital readmissions, emergency department visits, and healthcare expenses than patients without infection in a retrospective, observational cohort study [19]. This indicates a greater strain on the healthcare system, which is already plagued by financial constraints and expenses borne by the patient and his caregivers.

A total of 15 patients had plateauing/ incline in CRP levels on or after the third post-operative day, these patients had presented with clinical signs of infections such as local hyperthermia, fever, skin necrosis, and discharge from the surgical site. The discharge from the site was sent for isolation of organism and organisms isolated in all the samples were found to be of *Staphylococcus Aureus.* Isolation of organisms is a gold standard investigation in confirming postoperative wound infection. The patients with infection were started on parenteral antibiotics according to their culture & sensitivity report. The data collected in our study could be used to aid in the early detection of surgical infection, with the highest rise in CRP occurring on the third postoperative day in our study. 

One of the study's limitations was that we only evaluated CRP as a serum marker of postoperative infection. Although various blood markers such as interleukins reflect increased inflammatory activity, serum CRP is a sensitive and superior parameter for monitoring orthopaedic postoperative sequelae when compared to other cytokines [20]. Depending on the fracture type or the extent of surgical intervention may fluctuate even when using the same implant. These limitations make it difficult to extrapolate the findings of this investigation, and more multicentre prospective studies will be needed to confirm the findings' validity.

## Conclusions

C-reactive protein is a valuable diagnostic tool for detecting and monitoring postoperative wound infections. In our study, we conclude that the presence of fracture-related infection post-surgical intervention is indicated by an increase in C-reactive protein on the third and seventh postoperative days. The infection rates are higher in an emergency orthopaedic trauma surgery when compared to elective orthopaedic trauma surgery. Monitoring C-reactive protein levels aid in the earlier detection of complications, planning of a suitable intervention, and a better prognosis for patients with postoperative infections. Rather than expecting clinical manifestations to appear before commencing infection intervention, the rise of CRP on the third day after surgery could be considered signs of infection development. Timely identification of infection will aid in the management of complications that may emerge if the infection worsens or becomes unmanageable later.
